# Development and validation of the Pediatric-Youth Hyperphagia Assessment for Prader-Willi syndrome

**DOI:** 10.4178/epih.e2022014

**Published:** 2022-01-10

**Authors:** Sung Yoon Cho, Danbee Kang, Minji Im, Aram Yang, Min-Sun Kim, Jiyeon Kim, Eu-Seon Noh, Eun Kyung Kwon, Eujin Choi, Sunju Han, Young Ah Park, Min Jung Kwak, Youngha Kim, Juhee Cho, Dong-Kyu Jin

**Affiliations:** 1 Department of Pediatrics, Samsung Medical Center, Sungkyunkwan University School of Medicine, Seoul, Korea; 2 Department of Clinical Research Design and Evaluation, Samsung Advanced Institute for Health Science and Technology, Sungkyunkwan University School of Medicine, Seoul, Korea; 3 Center for Clinical Epidemiology, Samsung Medical Center, Sungkyunkwan University School of Medicine, Seoul, Korea; 4 Department of Pediatrics, Pusan National University Hospital, Pusan National University School of Medicine, Busan, Korea

**Keywords:** Prader-Willi syndrome, Hyperphagia, Appetite, Questionnaire, Eating disorder, Obesity

## Abstract

**OBJECTIVES:**

Hyperphagia is a highly stressful, life-threatening feature of Prader-Willi syndrome (PWS). It is important to assess this complex behavior accurately over time. This study aimed to develop and validate the Pediatric-Youth Hyperphagia Assessment for Prader-Willi syndrome (PYHAP) as a tool targeting children and adolescents.

**METHODS:**

After an extensive literature review and qualitative interviews, the final version of the PYHAP with 14 questions in 3 domains (verbal [5], behavior [4], and social [5]) was developed and tested at Samsung Medical Center in Seoul, Korea from July 2018 to September 2019. Exploratory factor analysis and confirmatory factor analysis (CFA) were performed to confirm construct validity. The correlations between the PYHAP and the Korean Children’s Eating Behavior Questionnaire (K-CEBQ) were calculated to evaluate convergent and discriminant validity. Criterion validity and the validity of the response categories were also tested.

**RESULTS:**

Cronbach’s alpha coefficient of the PYHAP was 0.91. The fit indices for CFA were good (comparative fit index, 0.87; standardized root mean squared residual, 0.08). The domains of the PYHAP were closely correlated with the relevant domains of the K-CEBQ. The accuracy of the PYHAP score for predicting uncontrolled hyperphagia was good (area under the curve, 0.75; 95% confidence interval, 0.65 to 0.85).

**CONCLUSIONS:**

The PYHAP was confirmed to be a reliable and valid tool to evaluate hyperphagia in children and adolescents with PWS via caregivers’ assessments. It is recommended to use the PYHAP to communicate with parents or caregivers about patients’ hyperphagia or to monitor and manage extreme behaviors in children with PWS.

## INTRODUCTION

Prader-Willi syndrome (PWS) is a genetic disorder that results from a lack of the expression of paternal alleles in the PWS region of chromosome 15q11-13. It has an incidence of 1 per 10,000-15,000 [1]. The hallmark of PWS is hyperphagia, which is defined as an incessant feeling of insatiable hunger, regardless of food intake. Patients with PWS exhibit poor feeding habits and failure to thrive until nine months of age, after which they tend to be obese due to hypothalamic pituitary dysregulation-induced hyperphagia with a lack of satiety. This can lead to severe obesity in childhood, which can progressively develop into type 2 diabetes mellitus and other metabolic disorders. This results in increased morbidity and mortality, as well as poor quality of life among children with PWS [2].

Hyperphagia is a highly stressful, life-threatening feature of PWS [3]. It is important to assess this complex behavior accurately over time; therefore, researchers have sought to find reliable methods to measure hyperphagia in PWS. The 16-item Food-Related Problems Questionnaire [4] was developed in 2003 to assess food preoccupation, satiety impairment, and related negative behaviors in individuals with PWS. This is a self-reported questionnaire based on adults who were living in PWS-designed group homes; therefore, it is unsuitable for children with PWS, who have different hyperphagia symptoms. In addition, it is unsuitable for children with PWS who have intellectual disabilities [5,6]. Dykens et al. [3] developed the Hyperphagia Questionnaire (HQ, 13 questions) that obtains responses from the parents and caregivers of children with PWS. This questionnaire sought to assess the maladaptive behaviors and compulsivity of children with PWS. The HQ was developed for individuals of any age; however, its validation study included a substantial proportion of adults. In PWS patients, hyperphagia is more pronounced in childhood and adolescence than in adulthood; therefore, previous tools might be unsuitable for evaluating hyperphagia in children or adolescents with PWS. In addition, no tool evaluating hyperphagia has been developed with Asian PWS patients. Therefore, we developed and validated the Pediatric-Youth Hyperphagia Assessment for Prader-Willi syndrome (PYHAP) to evaluate hyperphagia in children and adolescents with PWS in Korea.

## MATERIALS AND METHODS

### Instrument development

We established an expert group to develop this tool, which consisted of 2 pediatricians, 3 pediatric nurses, and 2 behavioral scientists. They performed an extensive literature review and confirmed that there was no existing instrument that specifically addressed hyperphagia in children and adolescents with PWS; the existing tools that can evaluate hyperphagia in children and adolescents with PWS were not specifically designed for this cohort whose problematic behaviors are different from adults.

Next, the expert team conducted qualitative interviews to develop the initial questions for this tool. Semi-structured in-depth interviews were conducted with 6 parents whose offspring were > 3 years of age and genetically confirmed to have PWS. Parents were asked how their children with PWS expressed themselves or behaved in relation to hyperphagia, including extreme behaviors. In addition, parents reported the daily living conditions, including family dynamics and eating environment, the patients’ eating behaviors, and other health behaviors that might be affected by hyperphagia, such as sleeping or exercise. Parents commonly mentioned that their children persistently expressed hunger and asked for food. Additionally, the children were easily upset when their requests for food were denied. Parents reported that children waited in places where food was stored and showed extreme behaviors, such as eating uncooked food or eating food in unsanitary places. Furthermore, parents reported that hyperphagia interfered with everyday life, as their children were found to eat secretly or take money for food without permission. In addition, parents expressed concerns that their children ate more and faster than other children.

### Instrument validation

#### Study participants

The main caregivers of children and adolescents with PWS were recruited at a pediatric outpatient clinic at Samsung Medical Center (SMC), Seoul, Korea between July 2018 and September 2019. Individuals were eligible to participate in this study if their children were aged 3 years or older, if they were the primary caregivers who lived with the patient, and could observe the daily activities of the patient. Participants were excluded if their children had been prescribed any psychiatric medication for > 6 months at the time of the survey.

#### Measurement

The PYHAP questionnaire, which included 16 questions in 3 domains (verbal [5], behavior [6], and social [5]), was developed to assess hyperphagia in children and adolescents with PWS based on the literature review and qualitative study. Next, a pilot test was performed with 3 parents, in which they were asked to complete the questionnaire, followed by an interview where they were provided feedback on its content. All participants reported that the questionnaire covered the aspects of hyperphagia among children and adolescents with PWS; however, they found two questions in the behavior domain confusing. These questions asked whether a child with PWS ate more or faster than other children. They found this difficult to answer because it was not clear what “other children” referred to (other children with PWS, children of the same age without PWS, or non-PWS siblings). The expert group agreed that these items might not be appropriate for assessing the hyperphagic behaviors of children and adolescents with PWS; therefore, these 2 items were excluded from the questionnaire. In addition, slight changes were made to improve clarity of the questionnaire.

The final version of the PYHAP included 14 questions in 3 domains (verbal [5], behavior [4], and social [5]). Responses were recorded using a 5-point Likert scale (1, never; 2, rarely; 3, sometimes; 4, often; and 5, always). The response scale for 4 questions that assessed extreme behaviors (eating uncooked food, eating food in unsanitary places, taking money for food, taking food without purchasing) were dichotomous (1, yes; 0, no). Total scores were calculated by summing the responses for all items. Higher scores indicated uncontrolled hyperphagia. In addition to the PYPHP, eating behaviors were assessed using the Korean Children’s Eating Behavior Questionnaire (K-CEBQ) [7] to evaluate concurrent and discriminant validity. The K-CEBQ is a 35-item parent-report questionnaire assessing children’s eating style. Eating style is assessed using 8 scales: food responsiveness (4 items), enjoyment of food (4 items), emotional overeating (4 items), desire to drink (3 items), satiety responsiveness (5 items), slowness in eating (4 items), and emotional undereating (4 items), and fussiness (7 items). Informants rate the frequency of their child’s behaviors and experiences on a 5-point scale (1, never; 2, rarely; 3, sometimes; 4, often; 5, always). Socio-demographic and clinical characteristics (current age, sex, and body mass index [BMI] for patients; age, sex, BMI, education, monthly family income, exercise, and family medical history for main caregivers) were recorded.

### Statistical analysis

Descriptive statistics were used to report participants’ characteristics. The mean and standard deviation (SD) was reported for each item of the PYHAP. Exploratory factor analysis (EFA) was performed to evaluate construct validity and determine the underlying structure of the PYHAP [8]. After extracting the factor structure, we performed confirmatory factor analysis (CFA) using the maximum likelihood method without missing values to test whether our factor structure fit the data [9]. Several goodness-of-fit indices were used to evaluate the model fit, including the comparative fit index (CFI) and standardized root mean squared residual (SRMR). A CFI > 0.9 and SRMR < 0.08 indicated a good fit to the data [10,11].

To examine convergent and discriminant validity, hypotheses on the direction and magnitude of Pearson correlations coefficients between the PYHAP and the K-CEBQ were formulated a *priori* [12]. We expected that the PYHAP would have positive correlations with emotional overeating, food responsiveness, and desire to drink domains in the K-CEBQ as indicators of convergent validity, and that it would have negative correlations with the satiety responsiveness, food fussiness, and slowness domains, as well as low or no correlation with the emotional undereating domain, among the eating domains of the K-CEBQ as indicators of discriminant validity. We used item response theory (IRT) to estimate the validity of the response categories. Item sets were calibrated by fitting to the Samejima graded response model (GRM). For each item, the GRM estimates a slope or discrimination parameter reflecting the degree of association of the item responses with the latent construct being measured, and 4 threshold parameters (for items with 5 response options, or 1 threshold for items with 2 response options) that indicate the level of hyperphagia at which a response in a given category or higher becomes probable. After item selection for the final pool, differential item functioning (DIF) was investigated between responses for younger (3-7 years old) versus older (≥ 8 years old) children. Logistic regression was performed to test for both uniform and non-uniform DIF, which indicated whether an item favored 1 group for all or only some of the latent trait values.

In addition, we calculated the area under the curve (AUC) to test criterion-related validity and used parent-reported uncontrolled hyperphagia as a gold standard. For the analysis, we asked parents “Are your child’s food-related behaviors controlled compared with other children of the same age?” using the 5-point Likert scale (with excellent, good, fair, poor, and very poor). Responses of “poor” and “very poor” were considered to indicate uncontrolled hyperphagia. We used the Youden index to identify cut-off values [13]. Total scores were calculated by summing the responses for 14 items. Based on the item threshold parameter in IRT, we scored a “yes” response as 4 points for extreme behavior items. Scores ranged from 14 to 66, with higher scores representing worse hyperphagia. We adjusted for age, sex, and BMI. The significance level was p-value< 0.05 (2-sided). All statistical analyses were performed using Stata version 15 (StataCorp., College Station, TX, USA).

### Ethics statement

All procedures were approved by the Institutional Review Board of SMC (IRB No. 2017-12-057) and all participants provided written informed consent.

## RESULTS

### Study participants

Eighty-seven main caregivers participated in the survey. The median (interquartile range, IQR) age of patients with PWS and their main caregivers was 10 years (IQR, 5-18) and 43 years (IQR, 38-50), respectively (Table 1).

### Construct validity: factor analysis

**Figure 1.**
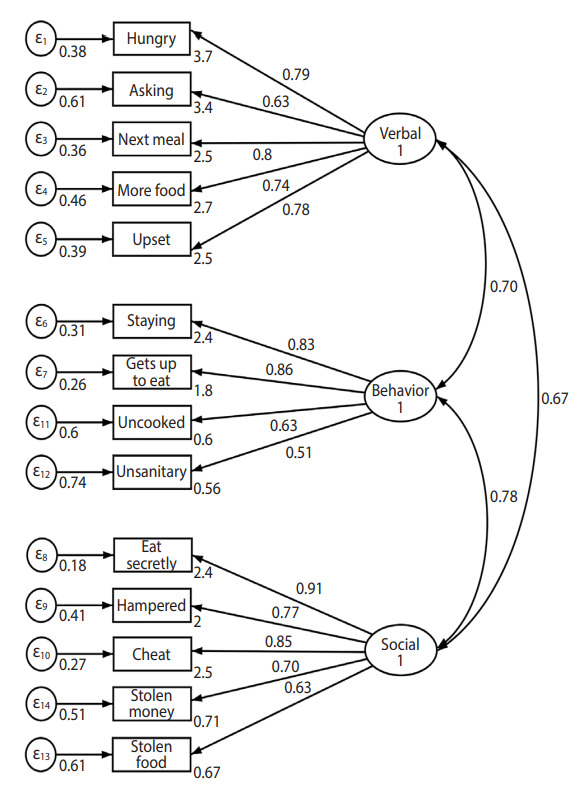
Structural equation model for confirmatory factor analysis.

**Figure 2.**
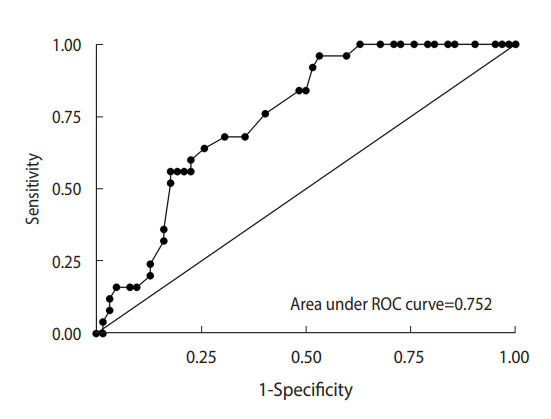
Receiver operating characteristic (ROC) curve to test criterion-related validity.

The EFA indicated a 3-factor solution with an eigenvalue > 1.0. The factor loadings for the three retained and varimax rotated factors are presented in Table 2. The variance explained by the 3-factor solution was 69%. “Eating food in unsanitary places” and “food cravings interfering with everyday life” had relatively low factor loading values; however, we did not exclude these items based on the expert opinion that these were important items to measure hyperphagia in PWS. Thus, the expert group confirmed 14 questions in 3 domains: verbal, behavior, and social. The Cronbach alpha coefficient of the PYHAP was 0.91, and the subdomains showed high internal reliability, ranging from 0.80 to 0.89 (Table 2). A further examination of the factor structure of the 14-item PYHAP was performed using CFA, which revealed high loading (0.51-0.91). The fit indices for this model were good (CFI= 0.87; SRMR= 0.08) (Figure 1). The verbal, behavior, and social subdomains were closely correlated (0.77) (Figure 2).

### Validity of the response categories

**Figure 3.**
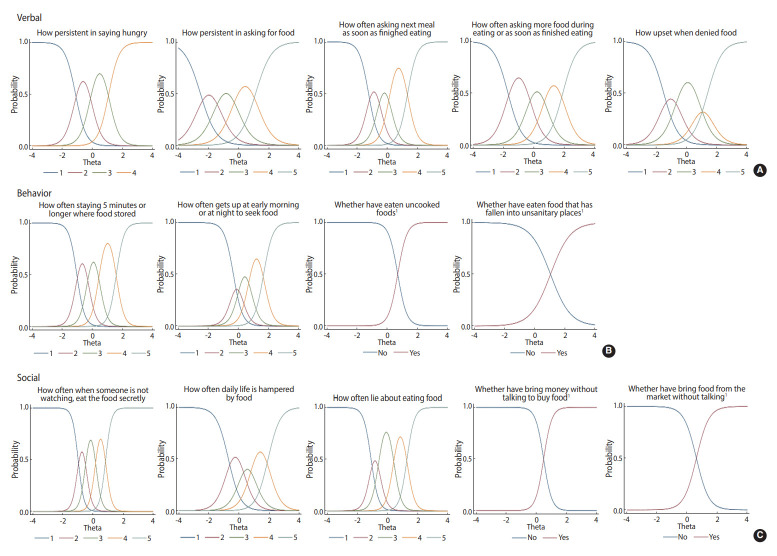
Item characteristics (A: verbal, B: behavior, and C: social) curve. Response options: 1, never; 2, rarely; 3, sometimes; 4, often; 5, always. 1Bivariate (no or yes).

The difficulty levels for each item increased as responses increased (Figure 3). The items covered a range on the item difficulty spectrum. The items “staying/lingering around where food is kept for more than 5 minutes” (p=0.03) and “getting up early in the morning or late at night to seek food” (p=0.02) showed uniform DIF. In addition, “eating food in unsanitary places” had a significantly uniform DIF by age (< 8 vs. ≥ 8 years; p<0.01).

### Convergent and discriminant validity

The verbal domain was significantly correlated with food responsiveness (r=0.63, p<0.001). The behavior domain was correlated with food responsiveness (r=0.57, p<0.001) and emotional overeating (r=0.63, p<0.001). The social domain was significantly correlated with enjoyment of food (r=0.54, p<0.001) and food responsiveness (r=0.53, p<0.001) (Table 3). The total items were positively correlated with enjoyment of food (r=0.59, p<0.001), emotional overeating (r=0.60, p<0.001), and food responsiveness (r=0.67, p<0.001), and were negatively correlated with satiety responsiveness (r=-0.43, p<0.001), food fussiness (r=-0.33, p=0.002), and slowness in eating (r=-0.38, p<0.001) (Table 3).

### Criterion-related validity

The questionnaire revealed that 28.7% of the children had uncontrolled hyperphagia. Furthermore, children with uncontrolled hyperphagia were more likely to have higher PYHAP scores than those without uncontrolled hyperphagia (44.4 vs. 36.5, p<0.01). The accuracy of the PYHAP score for predicting uncontrolled hyperphagia was characterized by an AUC of 0.75 (95% confidence interval [CI], 0.65 to 0.85 (Figure 2). The mean±SD PYHAP score of children with PWS was 37.3±11.9, and the cut-off value for uncontrolled hyperphagia was 39 out of 66. In total, 41.3% of the patients had uncontrolled hyperphagia.

## DISCUSSION

In this study, we found that the PYHAP was a reliable and valid tool to evaluate hyperphagia in children and adolescents with PWS via caregivers’ assessments. The 3 factors that emerged in this analysis were reflected in the 3 subdomains: verbal, behavioral, and social. These subdomains reflect the findings of the qualitative interviews. EFA and CFA confirmed the construct validity of the tool, and the response scale was also valid. Furthermore, concurrent and discriminant validity were demonstrated by its varying degrees of correlation with the K-CEBQ. Taken together, our results provide strong evidence supporting the construct validity and reliability of the PYHAP.

The PYHAP was easily administered and completed by the main caregivers of the children and adolescents with PWS. EFA and CFA confirmed our hypothesis regarding the underlying constructs of the PYHAP: verbal, behavior, and social domains. The themes of the 3 domains were consistent with previously identified problems related to hyperphagia among children and adolescents. The verbal subdomain represented the verbal and emotional expression of appetite. Children with PWS show anomalous behaviors that reflect hyperphagia even if they could not express their appetite in language. In particular, these behaviors might be better markers of hyperphagia in non-verbal children. The behavioral domain of the PYHAP included abnormal behaviors, such as staying where food is stored, getting up early in the morning or late at night to seek food, and eating uncooked or discarded foods, which are important appetite monitoring behaviors. Compared with the previous questionnaires, including the HQ [3,4], the PYHAP covers the social difficulties related to hyperphagia more comprehensively. Specifically, the PYHAP asks about children’s deviant behaviors such as lying about eating food and stealing food or money to purchase food, which often results in social problems.

In this study, we also conducted an IRT analysis, in which all items showed good discrimination. Higher values of this parameter are associated with items that are better able to discriminate between contiguous trait levels near the inflection point. This is consistent with the fact that fewer people responded “always” to these items than to any other items. This indicates that children who always performed the actions described in these verbal or behavioral domains were more likely to have uncontrolled hyperphagia than other children. Hyperphagia-related behavior increases with age; however, the hyperphagic drive remains stable, while uncontrolled hyperphagia is reduced in older adults [3]. These differences may be related to the ability to perform specific behaviors. Young patients may have limited behaviors based on their physical function. In addition, Hartley et al. [14] have reported that individuals with PWS aged 20-29 years had significantly higher levels of aggressive behavior scores than those aged 12-19 years and 30-45 years. In line with this, we found that the items related to whether children persistently expressed hunger or ate food in unsanitary places showed uniform DIF by age. DIF occurs when items in a measure perform in ways that are different for members of a target group and the different performance is not related to an individual’s overall ability being assessed [15]. This suggests that the PYHAP should be carefully assessed by age group.

When we compared the PYHAP to the K-CEBQ, there were positive correlations with the factors related to increased appetite, and negative correlations with factors related to decreased appetite in the K-CEBQ. In addition, this measure was found to be suitable for distinguishing between patients with and without uncontrolled hyperphagia (AUC, 0.88). When the frequency of hyperphagia-related behaviors was very high, even minor symptoms related to hyperphagia showed scores corresponding to extreme behaviors. Therefore, the frequency of words or actions should be carefully observed. The PYHAP can distinguish between differences in appetites among patients with PWS, and allow caregivers and clinicians to observe changes in the appetites of each patient objectively over time. This will help determine the type and timing of interventions required by patients with PWS.

This study has several limitations. First, the PYHAP was developed using Korean patients with PWS. Nonetheless, the previous literature revealed that PWS patients experience similar problems related to hyperphagia [16]. While additional validation research would be necessary, we believe that the PYHAP could be a reliable measure for PWS patients in other countries. Second, we did not conduct a test-retest analysis. Our study design did not include an evaluation to confirm clinical stability; however, the items included in the PYHAP were relatively objective measurements. Third, we did not assess non-food behavioral and emotional problems objectively in our PWS cohort. Further studies should be performed using tools to evaluate behavioral problems, emotion, and quality of life. In addition, further investigations into diet regimens and family environments should be performed in a larger number of patients with PWS.

Nonetheless, study has several strengths. We included a relatively large sample size of children and adolescents for a study with rare disease. Because the HQ was developed based on interviews with parents and caregivers of children and adolescents older than 8 years old, the HQ seemed not to be able to cover certain nonverbal or social behaviors of younger children, such as seeking food in the morning. In contrast, we included parents of PWS children who were 3 years or older in the development phase and onwards, and we covered issues related to both children and adolescents. In addition, we focused on patients with PWS who lived in their family home in order to obtain exact information from their main caregivers, who could observe and describe the behaviors of their children objectively and consistently. Furthermore, we conducted qualitative interviews before the quantitative test to ensure the content validity of the questionnaire.

All PWS patients need sustained and intensive dietary interventions once they are diagnosed with PWS [17]. In conclusion, the PYHAP offers a greater understanding of the natural course of eating behaviors and uncontrolled hyperphagia in children and adolescents with PWS. Health professionals are recommended to use the PYHAP as a tool to communicate with parents or caregivers about patients’ hyperphagia or to monitor and manage extreme behaviors of children with PWS. Clinical trials with appetite suppressants for PWS patients have recently been conducted. The PYHAP will be a useful tool to evaluate the efficacy of new treatments to control appetite in future clinical trials.

## Figures and Tables

**Table 1.** Characteristics of children and adolescents with PWS and their main caregivers

Characteristics	n (%)
Children and adolescents with PWS (n=87)	
Age, median (IQR)	10 (5-18)
Sex	
Male	43 (49.4)
Female	44 (50.6)
BMI (kg/m^2^), median (IQR)	23.8 (18.9-30.4)
Main caregivers (n=87)	
Age, median (IQR)	43 (38-50)
Sex	
Male	20 (23.3)
Female	66 (76.7)
BMI (kg/m^2^), median (IQR)	23.1 (20.8-25.2)
Monthly family income (US$)	
<3,000	24 (27.9)
≥3,000	60 (69.8)
Unknown	2 (2.3)
Education	
≤High school	25 (29.1)
≥College	61 (70.9)

PWS, Prader-Willi syndrome; SD, standard deviation; BMI, body mass index; IQR, interquartile range.

**Table 2.** Factor loadings and reliability of the PYHAP (n=87)

Items		Mean±SD	Factor loading values			Cronbach’s alpha coefficients
			1	2	3	
Verbal						0.86
	Persistently expressing hunger	3.5±1.0	0.80	-	-	
	Persistently asking for food	3.6±1.1	0.75	-	-	
	Asking for the next meal immediately after finishing a meal	3.1±1.2	0.83	-	-	
	Asking more food while eating or immediately after eating	2.8±1.1	0.71	-	-	
	Easily getting upset when their requests for food are denied	3.0±1.2	0.64	-	-	
Behavior						0.80
	Staying/lingering around where food is kept for more than 5 min	2.9±1.2	-	-	0.77	
	Getting up early in the morning or late at night to seek food	2.4±1.3	-	-	0.75	
	Eating uncooked foods^1^	1.8±1.3	-	-	0.76	
	Eating food in unsanitary places^1^	1.7±1.3	-	-	0.43	
Social						0.89
	Eating food secretly when someone is not watching	3.2±1.4	-	0.67	-	
	Food cravings interfering with everyday life	2.4±1.2	-	0.58	-	
	Lying about eating food	3.0±1.2	-	0.73	-	
	Taking money from others without telling them to buy food1	2.0±1.4	-	0.86	-	
	Taking food from stores without purchasing^1^	1.9±1.4	-	0.81	-	-
Total		37.3±11.9	-	-	-	0.91

PYHAP, Pediatric-Youth Hyperphagia Assessment for Prader-Willi syndrome; SD, standard deviation.

1 Bivariate (no or yes).

**Table 3.** Correlations of the PYHAP with the K-CEBQ (n=87)

Variables	Verbal		Behavior		Social		Total	
	r	p-value	r	p-value	r	p-value	r	p-value
Enjoyment of food	0.52	<0.001	0.44	<0.001	0.54	<0.001	0.59	<0.001
Emotional overeating	0.44	<0.001	0.63	<0.001	0.48	<0.001	0.60	<0.001
Food responsiveness	0.63	<0.001	0.57	<0.001	0.53	<0.001	0.67	<0.001
Satiety responsiveness	-0.42	<0.001	-0.25	0.020	-0.40	<0.001	-0.43	<0.001
Food fussiness	-0.18	0.090	-0.26	0.020	-0.39	<0.001	-0.33	0.002
Emotional undereating	-0.06	0.590	0.06	0.560	-0.23	0.030	-0.11	0.320
Slowness in eating	-0.32	0.002	-0.37	<0.001	-0.30	<0.01	-0.38	<0.001
Desire to drink	0.38	<0.001	0.37	<0.001	0.26	0.020	0.38	<0.001

PYHAP, Pediatric-Youth Hyperphagia Assessment for Prader-Willi syndrome; K-CEBQ, Korean Children’s Eating Behavior Questionnaire.
